# Ferroptosis-associated circular RNAs: Opportunities and challenges in the diagnosis and treatment of cancer

**DOI:** 10.3389/fcell.2023.1160381

**Published:** 2023-04-20

**Authors:** Ruotong Yang, Liwei Ma, Junhu Wan, Zhuofang Li, Zhengwu Yang, Zhuochen Zhao, Liang Ming

**Affiliations:** ^1^ Department of Clinical Laboratory, The First Affiliated Hospital of Zhengzhou University, Zhengzhou, China; ^2^ Key Clinical Laboratory of Henan Province, Zhengzhou, China

**Keywords:** ferroptosis, circular RNA, cancer, biomarker, signaling pathway

## Abstract

Ferroptosis is an emerging form of non-apoptotic regulated cell death which is different from cell death mechanisms such as autophagy, apoptosis and necrosis. It is characterized by iron-dependent lipid peroxide accumulation. Circular RNA (circRNA) is a newly studied evolutionarily conserved type of non-coding RNA with a covalent closed-loop structure. It exhibits universality, conservatism, stability and particularity. At present, the functions that have been studied and found include microRNA sponge, protein scaffold, transcription regulation, translation and production of peptides, etc. CircRNA can be used as a biomarker of tumors and is a hotspot in RNA biology research. Studies have shown that ferroptosis can participate in tumor regulation through the circRNA molecular pathway and then affect cancer progression, which may become a direction of cancer diagnosis and treatment in the future. This paper reviews the molecular biological mechanism of ferroptosis and the role of circular RNA in tumors and summarizes the circRNA related to ferroptosis in tumors, which may inspire research prospects for the precise prevention and treatment of cancer in the future.

## 1 Introduction

According to statistics, cancer mortality rates have been rising in the 20th century, and if cancer mortality rates continue to escalate, there will be an additional 3.2 million cancer deaths annually (Siegel et al., 2021). In 2020, breast cancer replaced lung cancer for the first time to become the world’s most diagnosed cancer. According to statistics, China accounts for 30% of all cancer deaths worldwide. Lung cancer remains the most common type of cancer in China and is the main cause of high cancer mortality. However, in terms of incidence based on gender, breast cancer is the most prevalent type of cancer among women. At present, China is undergoing a period of cancer transformation, and the burdens of lung cancer, gastrointestinal cancer and breast cancer are increasing (Cao et al., 2021).

Ferroptosis is an emerging form of cell death. The rapid development of cancer-related ferroptosis research provides new prospects for its application in cancer treatment. The main biochemical features of ferroptosis are metabolism of the xCT system, inhibition of L-Glutathione (GSH) and glutathione peroxidase 4 (GPX4), accumulation of iron ions and the occurrence of lipid peroxidation (Mou et al., 2019). Ferroptosis occurs through two main pathways: an exogenous or transporter-dependent pathway [(e.g., solute carrier family 7 member 11 (SLC7A11) and transferrin receptor (TRFC)] and an endogenous or enzyme-regulated pathway [(e.g., acyl-CoA synthetase long-chain family member (ACSL4) and apoptosis inducing factor mitochondria associated 2 (AIFM2)]. During the onset of ferroptosis, polyunsaturated fatty acids (PUFAs) are sensitive to both enzymatic and non-enzymatic oxidation (e.g., iron-dependent Fenton reaction) (Tang et al., 2021). In recent years, a growing number of studies have identified a key role for ferroptosis in cancer progression, such as in gastric cancer (Zhang et al., 2020), melanoma (Ubellacker et al., 2020), lung cancer (Lou et al., 2021; Wang et al., 2019), and pancreatic cancer (Ye et al., 2021).

A large number of studies have revealed the extent of systematic changes in RNA in cancer. Importantly, widespread changes in coding and non-coding RNAs (ncRNAs) [(e.g., microRNA (miRNA), long non-coding RNA (lncRNA), small nuclear RNA (snRNA) and circular RNA (circRNA)] in cancer affect many aspects of tumorigenesis (Goodall and Wickramasinghe, 2021). cCircRNAs have covalently closed structures and high stability and are involved in gene regulation. CircRNA exhibits a covalent closed-loop structure, which is produced by a process called reverse splicing in the pre-mRNA phase (Vo et al., 2019). CircRNA was initially considered splicing-related noise. In addition, circRNAs are tissue-specific and cell-specific, with cis-acting elements and trans-acting factors involved in the regulation of their production (Kristensen et al., 2019). Existing studies have found that circRNA regulates cancer progression by regulating miRNA expression or protein function in many cell processes by acting as a miRNA sponge (Yin et al., 2019), protein bait (Zang et al., 2020), or encoding peptide (Wu et al., 2020), or through another mode of action (Yin et al., 2019). CircRNAs are aberrantly expressed in cancer progression, and these aberrantly regulated circRNAs play key roles in promoting or suppressing tumors. In addition, due to their high stability, abundance and specific expression patterns, circRNAs may become biomarkers and therapeutic targets for cancer patients (Li et al., 2020a). Interestingly, ferroptosis is also involved in the regulation of cancer progression by circular RNA. Ferroptosis-related circRNAs may offer new directions for future cancer therapy (Xie and Guo, 2021). This review mainly discusses the mechanism of ferroptosis and circRNAs involved in cancer regulation, as well as the research prospects and biological significance of ferroptosis-associated circRNAs in future cancer early diagnosis and precision therapy.

## 2 Ferroptosis

**FIGURE 1:**
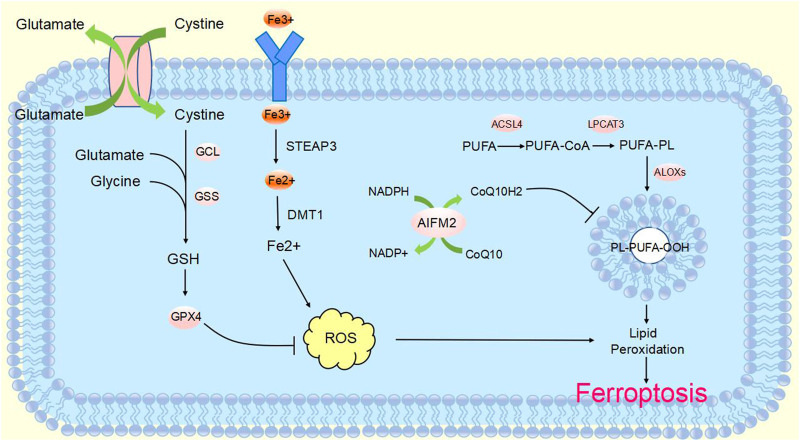
Molecular mechanisms of ferroptosisThe molecular mechanisms of ferroptosis can be roughly divided into three pathways. System xc-transports intracellular Glu to the extracellular space and extracellular Cys2 into the cell, which is then transformed into Cys for GSH synthesis. GPX4 ultimately reduces the accumulation of reactive oxygen species. The accumulation of lipid ROS leads to ferroptosis. Excess iron is the basis for ferroptosis execution. Circulated iron was combined with transferrin in the form of Fe3+, and then it entered into cells by TFR1. Iron in Fe3+ form was deoxidized to iron in Fe2+ by iron oxide reductase STEAP3. Ultimately, Fe2+ was released into a labile iron pool in the cytoplasm from the endosome mediated by DMT1, which led to ROS accumulation and eventual ferroptosis. ACSL4 and LPCAT3 promote the incorporation of PUFAs into phospholipids to form PUFA–PLs, which are vulnerable to free radical-initiated oxidation mediated by lipoxygenases (ALOXs). This ultimately causes lipid peroxidation and ferroptosis.

The definition of ferroptosis was first proposed by Dixon in 2012 and identifies it as an emerging mode of cell death (Dixon, 2017). Ferroptosis is mainly caused by high iron accumulation and lipid peroxidation. Iron-induced factors can affect glutathione peroxidase (GPx) levels through a variety of pathways, leading to elevated lipid reactive oxygen species (ROS) and a reduction in antioxidant capacity, ultimately leading to oxidative cell death (Li et al., 2020b). Ferroptosis has become a research hotspot in recent years. At present, the molecular mechanisms of ferroptosis are roughly divided into three pathways: deletion or activation of GPX4, iron metabolism and lipid peroxidation (Figure 1). Cysteine is introduced into the cell by the action of system xc-, while glutathione synthesis is supported by the action of glyoxylate carboligase (GCL) and glutathione synthetase (GSS) enzymes. At the same time, glutathione acts as a common substrate for GPX4, acting between the reduced and oxidized states. Inhibition of system xc- or GPX4 inactivation will result in lipid ROS accumulation and stimulate ferroptosis. The formation of ROS on lipids is mediated by hydrogen peroxide. PUFAs are incorporated into membrane PLs through the combined action of ACSL4 and lysophosphatidylcholine acyltransferase 3 (LPCAT3). Acyl-CoA synthetase long chain family member 3 (ACSL3) activates monounsaturated fatty acids (MUFAs), which compete with PUFAs for integration into PLs. Exogenous addition of MUFAs inhibits ferroptosis. In addition, lipophilic antioxidants and iron chelators may inhibit the occurrence of ferroptosis (Forcina and Dixon, 2019).

### 2.1 Mechanism of ferroptosis

#### 2.1.1 System xc- and GPX4 in ferroptosis

GPX4 is a selenoprotein originally discovered by Ursini that acts as a catalyst for the reduction of Phospholipid hydroperoxides (PLOOHs) in cells. GPX4 is a key molecule in the regulation of ferroptosis which functions mainly through the inhibition of lipid peroxidation. GPX4 converts GSH to oxidized glutathione (GSSG) and reduces toxic lipid peroxide (L-OOH) to alcohols (L-OH) (Li et al., 2020b). Direct inhibition of GPX4 or GSH synthesis can trigger ferroptosis, and glutathione is a key regulator of GPX4 (Wu et al., 2021). Inhibition of system xc-leads to depletion of GSH and inactivation of GPX4, which increases lipid peroxidation. The Fenton reaction of Fe2+ takes place, oxidizing lipids, producing large amounts of ROS and ultimately stimulating the onset of ferroptosis (Friedmann Angeli et al., 2014) (Figure 1). The level of cellular lipid peroxidation can be increased by lipoxygenase in addition to neutralizing GPX4-mediated lipid peroxidation. Lipoxygenase and GPX4 play opposite roles in ferroptosis. Lipid peroxidation is catalyzed by lipoxygenase, whereas GPX4 inhibits lipid peroxidation (Stockwell et al., 2020). Ding et al. (2021) found that the anti-TNBC effect of DMOCPTL mainly acts to induce GPX4 ubiquitination by directly binding to the GPX4 protein and then induce ferroptosis and apoptosis. This is the first report of ferroptosis induced by GPX4 ubiquitination. These findings reveal for the first time a new way to induce ferroptosis through GPX4 ubiquitination and provide ideas for us to further study the mechanism of ferroptosis.

#### 2.1.2 FSP1 in ferroptosis

AIFM2 was originally defined as a proapoptotic gene that resists ferroptosis caused by GPX4 deficiency. It was later named ferroptosis suppressor protein 1 (FSP1) (Doll et al., 2019; Wu et al., 2002). FSP1 functions as an oxidoreductase at the plasma membrane, reducing ubiquinone (also known as coenzyme Q10) to the lipophilic free radical scavenger ubiquinone, which limits the accumulation of lipid ROS in the membrane under conditions independent of the GPX4 pathway (Bebber et al., 2020; Dixon et al., 2012). Extramitochondrial ubiquinone, as a lipophilic radical-trapping antioxidant (RTA), is produced by FSP1 from CoQ10 via a reduction reaction and reduces lipid radical levels through the recycling of a-tocopherol (a-TTH) (Zheng and Conrad, 2020) (Figure 1). FSP1 functions synergistically with GPX4 inhibitors and has been shown to trigger ferroptosis in many cancers. FSP1 plays a key role in the non-mitochondrial antioxidant system independently of the common GSH-GPX4 pathway. This suggests the existence of a novel ferroptosis inhibitory pathway and that inhibition of FSP1 may render cancer patients more sensitive to ferroptosis-inducing chemotherapeutic agents, thereby affecting cancer progression (Bersuker et al., 2019).

#### 2.1.3 Iron in ferroptosis

Studies have found that excessive iron accumulation is a key cause of ferroptosis. Redox-active iron pools can catalyze the production of damaging free radicals from lipid peroxides via the Fenton reaction. Once lipid peroxide is not removed in time within the cell, increasing levels of lipid alkoxyl radicals will eventually trigger ferroptosis (Lu et al., 2017) (Figure 1). The name ferroptosis indicates that the process is iron dependent: intracellular iron levels are positively correlated with ferroptosis and can be inhibited by iron chelators. Transcriptional changes in key genes regulated by iron [(e.g., TFRC, iron responsive element binding protein 2 (IREB2) and ferritin heavy chain 1 (FTH1)] will affect the sensitivity to ferroptosis induced by erastin (Ma et al., 2016; Gao et al., 2015). Multiple metabolic pathways (e.g., enhanced iron uptake, reduced iron accumulation and inhibition of iron efflux) will result in increased iron levels and promote iron uptake through integrated signaling pathways in animal models (Chen et al., 2020). Iron produces a large amount of ROS, which may promote tumor development. However, iron-dependent accumulation of reactive oxygen species can also lead to ferroptosis, which can inhibit tumor cells. Some researchers have proposed a hypothesis to explain this contradiction, namely, that redox signaling of iron and thiols is in dynamic equilibrium in cancer cells to help cells resist ferroptosis, but this idea requires more evidence and further research (Toyokuni et al., 2017). To date, there are three known pathways involved in the iron-dependent accumulation of lipid ROS in iron-induced blepharoptosis. First, ROS are produced by iron through the Fenton reaction, which is a non-enzymatic inorganic chemical reaction. Second, the reactive oxygen species produced by lipid self-oxidation are controlled by iron-catalyzed enzymes. Third, reactive oxygen species are produced by the oxidation of arachidonic acid (AA) by iron-containing oxygenase. Iron metabolism plays an important role in ferroptosis, and its regulatory mechanism still needs to be further studied (Lei et al., 2019). Sun et al. (2015) found that heat shock protein beta-1 (HSPB1) could further downregulate intracellular iron content by reducing the level of telomeric DNA-binding factor (TRF1). Thus, high levels of HSPB1, a key gene in ferroptosis, could hinder the development of ferroptosis. In addition, the expression of IREB2, the main transcription factor that inhibits iron metabolism, can significantly increase the expression of ferritin light chain (FTL) and FTH1, thereby inhibiting erastin-induced ferroptosis (Gammella et al., 2015). In addition, Fang et al. found that iron released from heme degradation, which is regulated by the involvement of heme oxygenase-1 (HO-1), is closely associated with ferroptosis. However, there are conflicting data showing that HO-1 can promote and inhibit ferroptosis (Fang et al., 2020). In conclusion, iron metabolism regulates a variety of genes and molecular pathways, affects sensitivity to ferroptosis, and plays an important role in ferroptosis.

#### 2.1.4 Lipid peroxidation in ferroptosis

Lipid peroxidation also plays an important role in the progression of ferroptosis. Ferroptosis is triggered when an imbalance in the endogenous antioxidant system of the cell leads to massive accumulation of lipid ROS, causing toxicity and disruption of membrane structure. Thus, oxidative stress and cellular antioxidant levels are important regulators of lipid peroxidation that can induce ferroptosis (Kajarabille and Latunde-Dada, 2019). Oxidation and depletion of PUFAs may cause changes in membrane structure and fluidity, increasing membrane permeability and reducing membrane integrity. It is hypothesized that the thinning of the membrane and changes in curvature when ferroptosis occurs will reduce the stability of the membrane and ultimately lead to the formation of pores and micelles (Yang and Stockwell, 2016; Agmon et al., 2018; Snaebjornsson et al., 2020). Polyunsaturated fatty acid-containing phospholipid (PUFA-PL) oxidation has been demonstrated to stimulate ferroptosis. Genetic disruption of ACSL4 and LPCAT3 confirmed resistance to ferroptosis (Dixon et al., 2015). ACSL4 preferentially activates polyunsaturated fatty acids (such as AA) to incorporate lipids, while LPCAT3 preferentially incorporates polyunsaturated fatty acid coenzyme A into membrane phospholipids. Thus, deletion of ACSL4 and LPCAT3 can reduce the membrane burden of oxidizable PUFA-PLs and modulate ferroptosis (Shindou and Shimizu, 2009) (Figure 1). In addition to its typical function in the electron transport chain, CoQ10 can also “moonlight” with respect to lipophilic antioxidants. The loss of CoQ10 may induce or sensitize ferroptosis and participate in the regulation of ferroptosis to a certain extent (Shimada et al., 2016). Lysyl oxidase (LOX) also plays a role in the process of lipid peroxidation. In the absence of LOX enzyme activity, free intracellular iron is sufficient to catalyze Fenton chemistry on lipid peroxides to generate toxic hydroxyl or lipid alkoxy radicals to promote lipid peroxidation and ferroptosis (Kim et al., 2016). In addition, Sun et al. (2015) found that protein kinase C-mediated phosphorylation of HSPB1 could hinder lipid reactive oxygen species accumulation to counteract ferroptosis. Inhibition of the HSF1-HSPB1 pathway and phosphorylation of HSPB1 resulted in enhanced anticancer effects in xenograft mouse tumors. Phosphorylation of HSPB1 acts as an important inhibitor of ferroptosis, mainly by inhibiting iron uptake and lipid ROS accumulation. NFE2 like bZIP transcription factor 2 (NRF2) is also a key molecule in ferroptosis, and in tumor cells, NRF2 prevents the accumulation of lipid peroxides and lipid oxidation of target proteins for survival purposes. Although kelch like ECH associated protein 1 (KEAP1) can produce an anti-ferroptosis effect by activating NRF2, many active lipids are in turn involved in inhibiting the function of NRF2 target genes, which in turn cannot be ignored in the ferroptosis cascade response (Dodson et al., 2019). In addition, GPX4 acts as an antioxidant enzyme, and its stimulation or inactivation can also modulate ferroptosis by affecting lipid peroxidation. Glutathione, a coregulator of GPX4, prevents peroxidation of cells and membranes, thereby neutralizing lipid peroxidation and ensuring membrane fluidity. Inhibition of GPX4 leads to increased levels of ROS, while overexpression of GPX4 downregulates ROS, thereby inhibiting the onset of ferroptosis (Ribas et al., 2014; Yang et al., 2014; Su et al., 2019). The key mechanisms of ferroptosis are iron accumulation, lipid peroxidation, and GPX4 regulation. The mechanisms of ferroptosis are very complex, and more details need to be explored in depth.

## 3 Function of CircRNA in cancer

**FIGURE 2:**
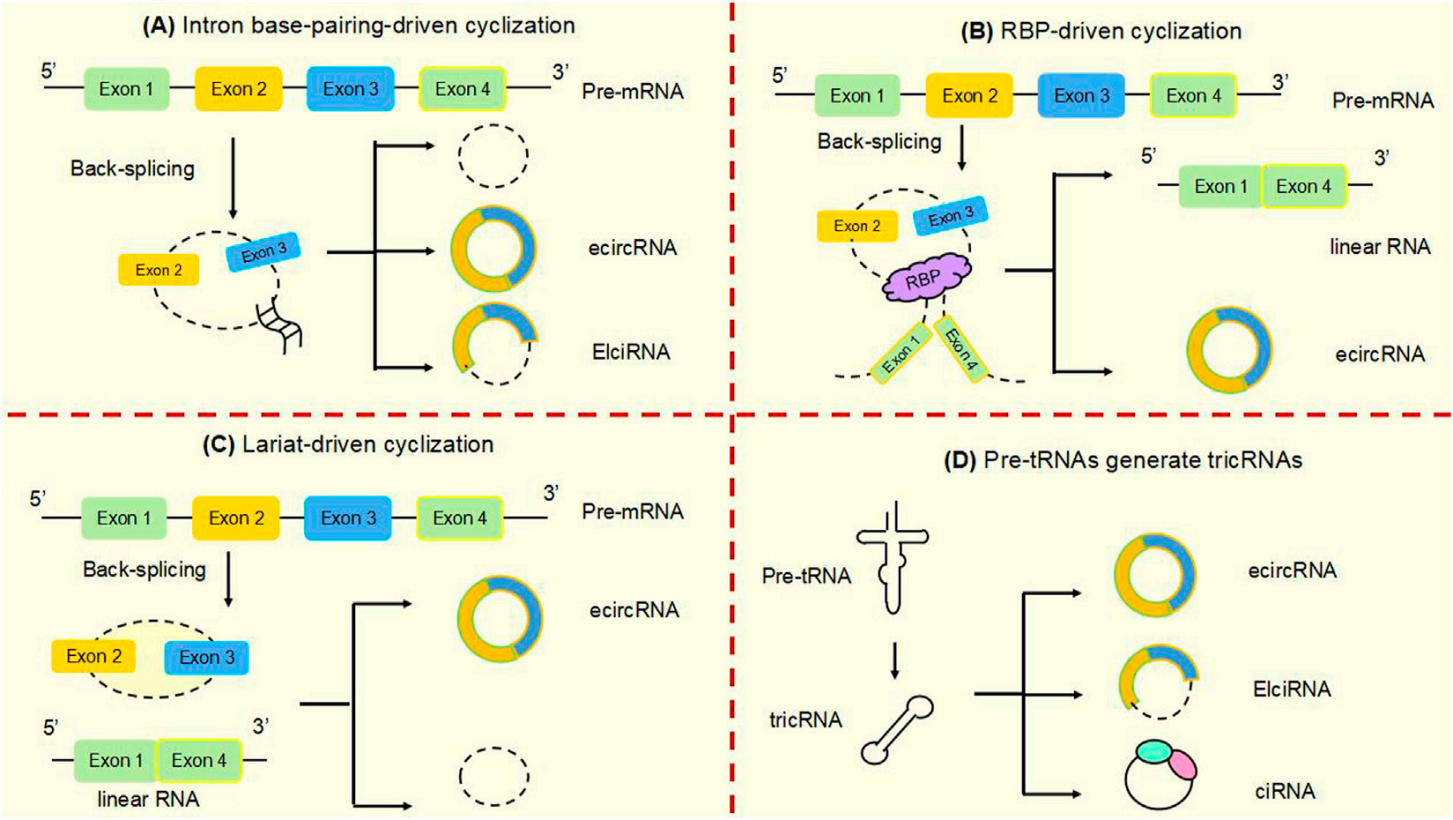
Biogenesis of circular RNAsCircRNAs are mainly produced from primary transcripts by back-splicing. The biogenesis of cyclic RNA is generated by four main mechanisms: **(A)** Intron base-pairing driven cyclization. Flanking intron complementation occurs by base pairing, allowing splice sites to join and drive cyclization. **(B)** RBP-driven cyclization. RBPs drive the cyclization of pre-mRNAs by binding to introns flanking introns in linear transcripts, leading to closer proximity of the splice site. **(C)** Lariat-driven cyclization. Back-splicing forms a lasso containing introns and skipped exons. Introns are spliced and subsequently form ecircRNA. **(D)** Pre-tRNA produces tricRNA. Pre-tRNA containing introns is sheared to produce triple RNA, then intron ends join and form triple RNA.

CircRNA is a non-coding RNA that is widely found in eukaryotes. It was originally defined as a product of abnormal RNA splicing and was also referred to as “transcriptional noise”. However, with the development of high-throughput genomic technologies, circular RNA can be studied in depth (Nisar et al., 2021). CircRNAs are made from pre-mRNA by back-splicing, where the downstream 3′splice site is linked to the upstream 5′splice site, which in turn is cyclized to form a covalent closed loop. They can be formed from introns or one or more exons. In addition, they are not susceptible to degradation by exonucleases, as they lack 3′and 5′ends (Zhang et al., 2014; Fontemaggi et al., 2021) (Figure 2). CircRNA biogenesis is mediated by four main mechanisms: intron base-pairing-driven cyclization (Figure 2A), RBP-driven cyclization (Figure 2B), lariat-driven cyclization (Figure 2C), and Pre-tRNAs generate tricRNAs (Figure 2D) (Wang et al., 2021a; Hu et al., 2023; Harper et al., 2021). Circular RNAs are classified into three main types according to their constituents: ciRNA, EIciRNA and exonic circRNA, which account for 85% of circular RNAs (Fontemaggi et al., 2021; Eger et al., 2018).

An increasing number of studies have shown that specific circular RNAs have the characteristics of carcinogenesis or tumor inhibition in the context of human cancers including gastric cancer (Zhang et al., 2019), non-small cell lung cancer (Chen et al., 2019a), breast cancer (Wang et al., 2018), bladder cancer (Cheng et al., 2021), and esophageal cancer (Meng et al., 2021). The specific expression and high stability of circRNAs in cells makes them a potential new target in cancer therapy (Fontemaggi et al., 2021). Therefore, further understanding of the biological functions and roles of circRNAs in different cancers, as well as the detection of signaling pathways, is important for the early diagnosis and precise treatment of cancer.

### 3.1 Role of CircRNA as MiRNA sponge in cancer

**FIGURE 3:**
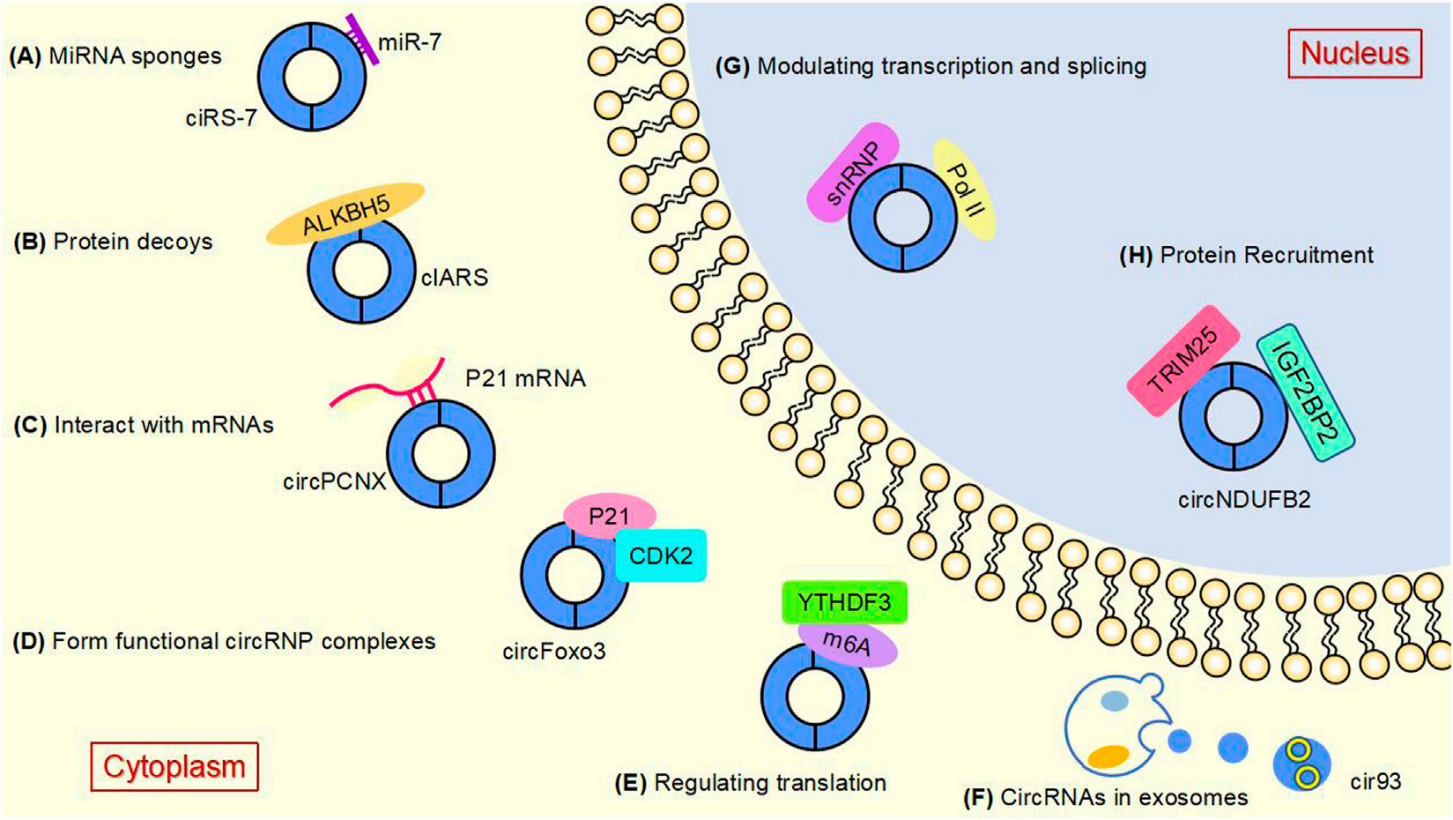
The main functions of circular RNAsAt present, several well-known functions of circular RNAs are as follows. **(A)** MiRNA sponges. CircRNAs can act as miRNA molecule sponges to prevent their binding to mRNA targets, which is the most widely studied function of circRNAs. **(B)** Protein decoys. CircRNAs can regulate the function of proteins by working as protein decoys or scaffolds. **(C)** Interact with mRNAs. CircRNA can directly bind mRNA, enhance mRNA stability and regulate gene expression. **(D)** Form functional circRNP complexes. CircRNA can bind proteins to form circRNP complexes that regulate downstream signaling pathways. **(E)** Regulating translation. CircRNAs can regulate translation, and m6A-mediated and internal ribosome entry sites (IRES) are the main mechanisms by which circRNAs initiate translation. **(F)** CircRNAs in exosomes. CircRNAs can be loaded into exosomes, resulting in exo-circRNAs that function as messenger RNAs for cellular communication by transferring them to recipient cells or neighboring cells. **(G)** Modulating transcription and splicing. CircRNA interacts with Pol II and snRNP, thereby regulating the transcription and splicing of parental genes. **(H)** Protein Recruitment. CircRNA can recruit proteins to specific motifs, promote protein assembly, and regulate gene expression.

Studies have shown that circular RNAs can act as miRNA sponges and gene transcripts and that interactions between circRNAs and proteins regulate gene expression. Moreover, circRNA can be translated into protein. Many previous studies have found that circRNA disorders manifest different functions and roles in cancer (Figure 3). However, the most widely studied is the role of miRNA sponge. CircRNA ciRS-7 was the first circRNA identified as a miR sponge, containing more than 70 miR-7 binding sites, ciRS-7 expression significantly repressed miR-7, resulting in reduced miR-7 activity and increased levels of miR-7-targeted transcripts, acting as a miR-7 inhibitor (Hansen et al., 2013a; Hansen et al., 2013b) (Figure 3A). found that circNRIP1, a sponge for miR-595, inhibited the oncogenic effects of esophageal cancer cell growth, migration and invasion through competitive binding of circNRIP1 and miR-595 to target the semaphorin 4D (SEMA4D) and PI3K/Akt signaling pathways in esophageal cancer cells (Zhou et al., 2021). Similarly, in hepatocellular carcinoma, circFBLIM1 can act as a competitive endogenous RNA (ceRNA) to regulate filamin binding LIM protein 1 (FBLIM1) expression levels through the sponge miR-346. CircFBLIM1 may become a biomarker and therapeutic target for hepatocellular carcinoma (Bai et al., 2018). Chen et al. also observed this function of circular RNA in bladder urothelial carcinoma. CircPRMT5 can absorb miR-30c by acting as a sponge and promote EMT in Urinary bladder tumor cells. CircPRMT5 is a key regulator in promoting EMT and invasiveness in UCB cells. Taken together, a wealth of experiments validate that circRNAs can act as miRNA sponges in competitively binding downstream target genes to influence cancer progression: this is one of the most common functions of circRNAs.

### 3.2 Interaction between CircRNA and RBP regulates cancer development

RNA binding proteins (RBPs) are a class of proteins that specifically recognize RNA binding regions and bind to each other (Tang and Hann, 2020). RBPs are widely involved in gene transcription and translation and control the production, maturation, localization, translation and degradation of cellular RNA (Okholm et al., 2020; Dominguez et al., 2018). As circular RNAs have been studied in depth, an increasing number of studies have confirmed that circRNAs can interact with RBPs to regulate cancer progression.

Circ-cux1 can promote aerobic glycolysis and increase the invasiveness of neuroblastoma cells by interacting with EWS RNA binding proteins. It also promotes association with myc-associated zinc finger protein (MAS) and stimulates enhanced transcriptional activity of MAS, suggesting that circ-cux1 is involved in regulating neuroblastoma progression by targeting myc associated zinc finger protein (MAZ) through binding to EWS RNA binding protein 1 (EWSR1) (Li et al., 2019). Furthermore, in lung adenocarcinoma, circdcun1d4 can bind the HuR protein, which elevates HuR activity, and acts as a scaffold to interact with thioredoxin interacting protein (TXNIP) mRNA, increasing the stability of TXNIP mRNA, which then plays a protective role in lung adenocarcinoma (Liang et al., 2021). reported that circnsun2 binds to insulin like growth factor 2 mRNA binding protein 2 (IGF2BP2) to target high mobility group AT-hook 2 (HMGA2), forming the circnsun2/IGF2BP2/HMGA2 RNA protein ternary complex, which is involved in promoting colorectal carcinoma formation (Chen et al., 2019b). Lu et al. (2020) found that heterogeneous ribonucleoprotein L (HnRNP-L) is a multifunctional RBP and that overexpression of HnRNP-L is involved in upregulating circCSPP1 in combination with miR-520h to target early growth response 1 (EGR1), promoting proliferation and apoptosis of prostate tumor cells and leading to increased progression of prostate cancer (Lu et al., 2021). HuR is a widely studied RBP. found that cIARS can interact with alkB homolog 5, RNA demethylase (ALKBH5) to act as a negative regulator in hepatocellular carcinoma and that downregulation of ALKBH5 significantly inhibited si-CIARS-induced iron deposition events, autophagic flux and ferritin phagocytosis (Liu et al., 2020) (Figure 3B). In conclusion, these findings suggest that circRNAs, as RBP sponges, interact with specific protein subunits to affect cancer progression, and more mechanisms need to be found.

### 3.3 Regulation of mRNA stability

CircRNAs have variety of biological functions and their functions as miRNA sponges and protein scaffolds interacting with RBPs have been extensively studied. In addition, circRNAs can directly bind mRNAs to affect gene expression levels, and regulate mRNA stability. CircRNA zinc finger protein 609 (circZNF609) has a role in regulating mRNA stability. Previous studies have shown that circZNF609 is a circRNA capable of promoting myogenic cell proliferation and regulating the malignant progression of rhabdomyosarcoma (RMS), a childhood skeletal muscle malignancy (Rossi et al., 2019). CircZNF609 was found by Rossi et al. to regulate cytoskeleton-associated protein 5 (CKAP5) mRNA stability and mediate tumor progression. Specifically, circZNF609 has multiple binding sites in ELAV-like RNA binding protein 1 (ELAVL1) (Beltran et al., 2022). CircZNF609 interacts with CKAP5 transcripts to promote ELAVL1 binding to mRNA, stimulate mRNA levels and translation, enhance mRNA stability and lead to changes in microtubule (MT) dynamics, thereby induce tumor cell proliferation.

Similarly, in cervical cancer cells, hsa circ0032434 (circPCNX) also plays a role in regulating mRNA stability. CircPCNX is an abundant target of F-box family protein (AUF1). Overexpression of circPCNX specifically blocks the association of AUF1 with p21 (CDKN1A) mRNA, thereby enhancing the stability of p21 mRNA and increasing the level of p21, which acts as a major inhibitor of cell proliferation, inhibiting the proliferation of cervical cancer cells and impeding the malignant development of cervical cancer (Tsitsipatis et al., 2021) (Figure 3C).

### 3.4 Role of functional circRNP complexes

CircRNA can also form functional circRNP complexes that regulate downstream signaling pathways and influence tumorigenesis. CircFoxo3 can affect tumor proliferation by regulating cyclins. CircFoxo3 forms the circFoxo3-p21-CDK2 ternary complex by binding to the cell cycle protein cell cycle protein-dependent kinase 2 (CDK2) and cell cycle protein kinase inhibitor 1 (p21), which cuts off the transition from G1 to S phase and eliminates the effect of p21 on the inhibitory effect of p21 on the cyclin A/CDK2 complex causes cells to stagnate in G1 phase and fail to transition to S phase, preventing normal cell cycle progression. CircFoxo3 interacts with p21 and CDK2 to form a functional circRNP complex that affects tumor cell proliferation and may be a direction for cancer therapy (Du et al., 2016) (Figure 3D).

CircRNA SCAR forms a SCAR-ATP synthase, H+ transporting mitochondrial F1 complex, beta subunit (ATP5B) scaffold in the mPTP complex, and ATP5B is a regulator of the mitochondrial permeability transition pore (mPTP) complex to block mPTP opening and inhibit mROS export to maintain mROS homeostasis *in vivo*. CircSCAR overexpression inhibits mROS in non-alcoholic steatohepatitis (NASH) generation and eliminates mROS export after lipid processing. This mitochondrial circRNA forms a functional circRNP complex that may be a therapeutic target for NASH (Zhao et al., 2020).

### 3.5 Role of circRNA in regulating translation

In addition to the functions of circRNAs described above, circRNAs can also regulate the translation of proteins or polypeptides. N6-methyladenosine (m6A)-mediated and internal ribosome entry sites (IRES) are the main mechanisms by which circRNAs initiate translation. The most abundant base modification of m6A RNA promotes efficient initiation of circRNA protein translation in human cells. M6A-driven translation requires the initiation factor eukaryotic translation initiation factor 4 gamma 2 (EIF4G2) and the m6A reader YTH N6-methyladenosine RNA binding protein F3 (YTHDF3) and is enhanced by the methyltransferase methyltransferase 3, N6-adenosine-methyltransferase complex catalytic subunit (METTL3), inhibited by the demethylase alpha-ketoglutarate dependent dioxygenase (FTO), and upregulated in response to heat shock (Hu et al., 2023; Yang et al., 2017; Lei et al., 2020) (Figure 3E). In the cytoplasm, the m6A-binding protein YTHDF1 promotes the translation of m6A-modified mRNA, and YTHDF2 accelerates the decay of m6A-modified transcripts (Shi et al., 2017). Song et al. (2022) found that eukaryotic translation initiation factor 3 subunit j (eIF3j) inhibits the translation process by inducing the dissociation of the eIF3 complex from circSfl. The C-terminal of eIF3j and the RNA regulators in the circS fluoride translation region (UTR) are key factors in the inhibition of eIF3j, and reveal the physiological relevance of eIF3j mediated circSfl translation inhibition to heat shock response. This provides a new direction for the cap less translation regulation of eIF3 and the involvement of circRNA in regulating translation.

### 3.6 Exosome-circRNAs (exo-circRNAs)

Exosomes play the role of messengers for intercellular communication. CircRNAs can be wrapped into exosomes to form exocytotic circRNAs that communicate by transferring to recipient cells or adjacent cells (Bai et al., 2019). The exosome circRNA_101093 (cir93) maintains elevated levels of intracellular cir93 in lung adenocarcinoma to regulate AA and inhibit the onset of ferroptosis. Exosomes and cir93 play an important role in the development of ferroptosis in lung adenocarcinoma, and blocking exosomes may be an approach to lung adenocarcinoma treatment (Zhang et al., 2022a) (Figure 3F). In colorectal cancer, circLPAR1 was detected as loaded in exosomes and its expression was reduced in plasma exosomes but recovered after surgical treatment. Exosomal circLPAR1 was internalized and direct binding of eIF3h by exosomal circLPAR1 blocked METTL3-eIF3h binding, inhibited translation of oncogene bromodomain containing 4 (BRD4), and reduced malignant progression of colorectal cancer (Zheng and Conrad, 2020). Exosomal circRNA has been shown to have important functions in cancer, and further mechanisms await our discovery.

### 3.7 Modulating transcription and splicing

Besides to their roles in the cytoplasm, circRNAs are also key factors in the regulation of transcription and splicing in the nucleus. Some circRNAs are now known to interact with RNA polymerase II (Pol II), thereby regulating the transcription and splicing of parental genes. Li et al. (2015) demonstrated that in the nucleolus, circRNAs can regulate the expression of nuclear genes, where EIciRNAs promote the transcription of parental genes in a cis manner by interacting with u U1 small nuclear ribonucleoprotein (snRNP) or binding to the Pol II promoter (Figure 3G). Ciankrd52, as a ciRNA, has a stronger R-loop formation capacity than its homologous pre-mRNA. Pre-mRNA is released from the R-loop by ciankrd52 substitution and ciRNA is removed by RNase H1 to achieve significant transcriptional elongation. This R-loop-dependent ciRNA degradation represses ciRNA levels by recruiting RNase H1 on the one hand, and resolves R-loop transcriptional elongation at some GC-rich ciRNA-producing motifs on the other (Li et al., 2021a).

CircRNA can also regulate gene shearing. SEPALATA3 (SEP3) is a homologous MADS box TF required for the homozygous heterozygous phenotype of Arabidopsis flowers. Exon 6 of SEP3 generates circSEP3 by reverse splicing (Conn et al., 2017). loop, whereas the linear RNA equivalent binds weakly to DNA in comparison. The production of R-loop represses transcription, splicing factor recruitment and AS2-4 are consistent. Taken together, this suggests that circRNAs may form R-loops more efficiently than linear RNAs to regulate gene transcription and splicing (Liu et al., 2022a).

### 3.8 Protein Recruitment

CircRNA interacts with proteins in a variety of ways. On the one hand, circRNAs act as protein scaffolds and facilitate the interactions between enzymes and substrates. On the other hand, circRNAs can also recruit proteins to specific motifs and stimulate protein assembly. CircMbl, a circular RNA with the first demonstrated protein sponge function, is involved in regulating the feedback pathway between the multifunctional proteotoxic mushroom (MBL) and circMb1. MBL promotes reverse shearing of circMbl by binding to a circMbl flanking intron containing a conserved MBL binding site. This suggests that the level of MBL can regulate the production of circMbl (Ashwal-Fluss et al., 2014).

Moreover, circNDUFB2 also functions as a protein scaffold, facilitating the interaction of tripartite motif containing 25 (TRIM25) and IGF2BPs to form a TRIM25/circNDUFB2/IGF2BPs ternary complex that induces ubiquitination and degradation of IGF2BPs, an effect enhanced by m6A modification of circNDUFB2. CircNDUFB2 regulates the development of non-small cell lung cancer by regulating the ubiquitination and degradation of IGF2BP2, confirming its role as a protein scaffold (Li et al., 2021b) (Figure 3H).

## 4 Ferroptosis related CircRNA involvement in regulating cancer progression

**FIGURE 4:**
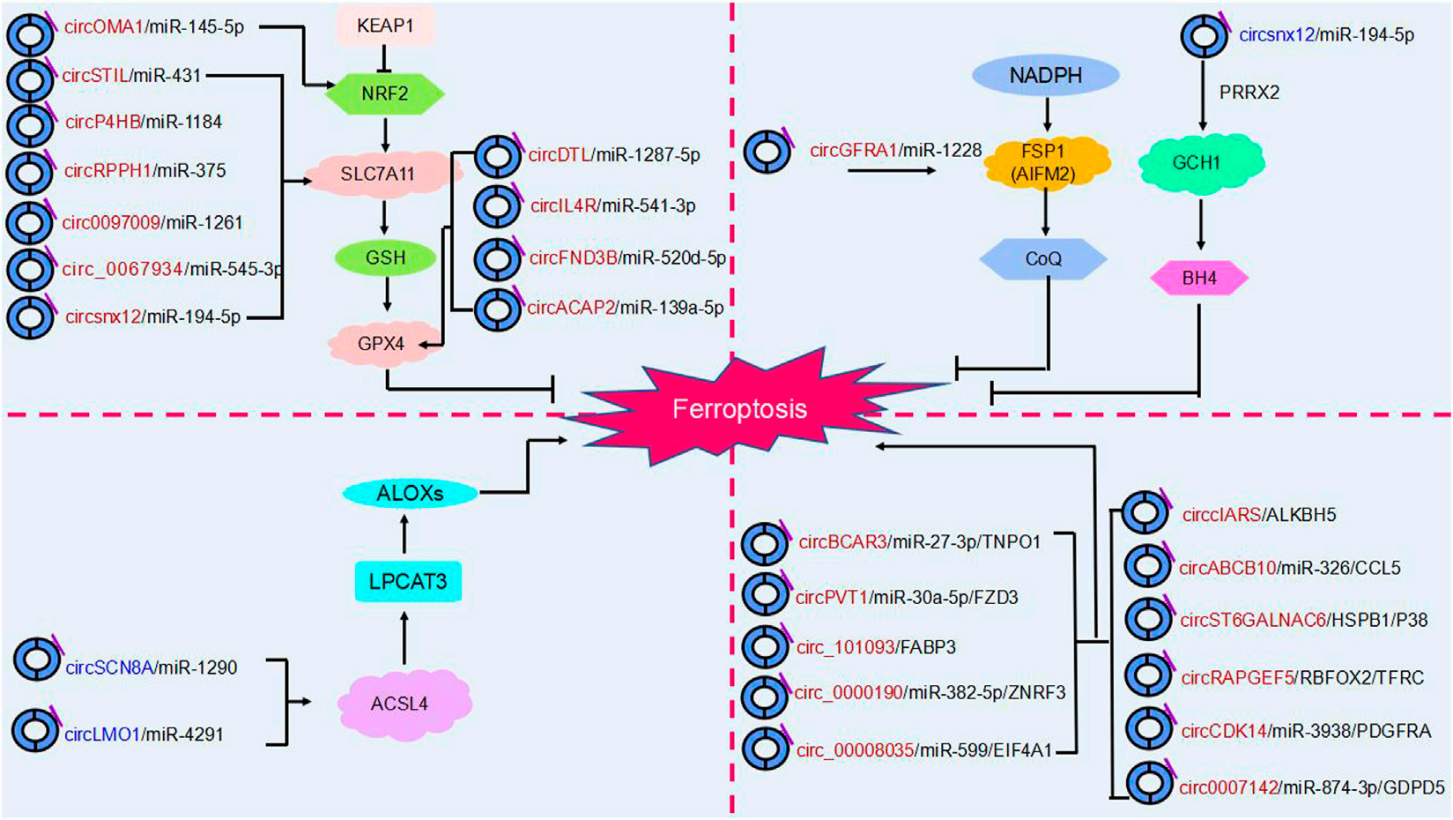
The regulatory role of circRNAs in ferroptosis. Ferroptosis is mainly regulated by four molecular pathways: the glutathione pathway, the ubiquinone pathway (NADPH- FSP1-CoQ10]], the tetrahydrobiopterin (GCH1- BH4] pathway and (ACSL4- LPCAT3- PUFA-PLs). As shown in the figure, circRNAs are involved in cancer progression by participating in four major molecular pathways regulating ferroptosis. Red circRNAs are upregulated in cancers, and blue circRNAs are downregulated in cancers.

In addition to the fact that circRNAs can influence cancer progression by regulating cell death (e.g., cell autophagy, apoptosis), recent studies have confirmed that circular RNAs can promote or inhibit ferroptosis by regulating key ferroptosis proteins after transcription and regulate tumor progression in various cancers through different molecular pathways. Thus, ferroptosis involving circRNAs may become a novel approach for cancer therapy (Xie and Guo, 2021) (Table 1) (Figure 4).

**TABLE 1:** Circular RNAs associated with ferroptosis in cancer.

CircRNA	Tumor type	Up/downregulation	Targeting miRNA	Targeting gene	Function	References
circFND3B	Oral cancer squamous cell carcinoma	up	miR-520d-5p	GPX4 SLC7A11	Ferroptosis, Apoptosis, Proliferation	Yang et al. (2021)
circ_0067934	Thyroid cancer	up	miR-545-3p	SLC7A11	Ferroptosis, Proliferation	Wang et al. (2021b)
circBCAR3	Esophageal cancer	up	miR-27a-3p	TNPO1	Ferroptosis, Proliferation, Invasion, Migration	Xi et al. (2022)
circPVT1	Esophageal cancer	up	miR-30a-5p	FZD3	Ferroptosis, Chemosensitivity	Yao et al. (2021)
circDTL	Non-small cell lung cancer	up	miR-1287-5p	GPX4	Ferroptosis, Apoptosis	Shanshan et al. (2021)
CircSCN8A	Non-small cell lung cancer	down	miR-1290	ACSL4	Ferroptosis, Proliferation, Invasion, Migration, EMT	Liu et al. (2023)
circP4HB	Lung adenocarcinoma	up	miR-1184	SLC7A11	Ferroptosis, Proliferation	Pan et al. (2022)
circRNA_101,093	Lung adenocarcinoma	up	_	FABP3	Ferroptosis	Zhang et al. (2022a)
circ_0000190	Gastric cancer	up	miR-382-5p	ZNRF3	Ferroptosis, Proliferation, Invasion, Migration	Jiang et al. (2022a)
circ_00008035	Gastric cancer	up	miR-599	EIF4A1	Ferroptosis, Apoptosis, Proliferation	Li et al. (2020)
circRPPH1	Gastric cancer	up	miR-375	SLC7A11	Ferroptosis, Proliferation	Liu et al. (2023)
circGFRA1	Breast cancer	up	miR-1228	AIFM2	Ferroptosis, Proliferation, Invasion, Metastasis	Bazhabayi et al. (2021)
circOMA1	prolactinoma	up	miR-145-5p	NRF2 GPX4	Ferroptosis, Proliferation	Wu et al. (2023)
circcIARS	Hepatocellular carcinoma	up	**_**	ALKBH5	Ferroptosis, Autophagy	Liu et al. (2020)
circ0097009	Hepatocellular carcinoma	up	miR-1261	SLC7A11	Ferroptosis, Proliferation, Invasion	Lyu et al. (2021)
circIL4R	Hepatocellular carcinoma	up	miR-541-3p	GPX4	Ferroptosis, Apoptosis, Proliferation	Xu et al. (2020)
circ0007142	Colorectal cancer	up	miR- 874- 3p	GDPD5	Ferroptosis, Proliferation, Apoptosis	Wang et al. (2021c)
circSTIL	Colorectal cancer	up	miR-431	SLC7A11	Ferroptosis, Proliferation	Li et al. (2023)
circABCB10	Rectal cancer	up	miR-326	CCL5	Ferroptosis, Apoptosis	Xian et al. (2020)
circLMO1	Cervical cancer	down	miR-4291	ACSL4	Ferroptosis, Proliferation, Invasion, Metastasis	Ou et al. (2022)
circACAP2	Cervical cancer	up	miR-193a-5p	GPX4	Ferroptosis, Proliferation	Liu et al. (2022b)
circSnx12	Ovarian cancer	up	miR-194-5p	SLC7A11	Ferroptosis, Chemoresistance	Qin et al. (2023)
bcircST6GALNAC6	Bladder cancer	up	_	HSPB1/p38	Ferroptosis	Wang S et al. (2022)
circRAPGEF5	Endometrial cancer	up	_	RBFOX2 TFRC	Ferroptosis, Proliferation	Zhang et al. (2022b)
circCDK14	Glioma	up	miR-3938	PDGFRA	Ferroptosis, Proliferation, Invasion, Migration, Tumorigenesis	Chen et al. (2022)
circLRFN5	Glioma	down	_	PRRX2, GCH1	Ferroptosis, Cell viabilities, Proliferation, Neurospheres formation, Stemness, Tumorigenesis	Jiang et al. (2022b)

### 4.1 Oral squamous cell carcinoma

The role of circFNDC3B in oral squamous cell carcinoma has been demonstrated. CircFNDC3B acts as a pro-oncogene in oral squamous cell carcinoma. It was found that knockdown of circFNDC3B resulted in elevated Fe2+, ROS accumulation, and downregulation of GPX4 and SLC7A11 expression levels, which stimulated ferroptosis and inhibited oral squamous cell carcinoma development. CircFNDC3B acts as a ceRNA binding miR-520d-5p to regulate SLC7A11. Overexpression of SLC7A11 reversed the ferroptosis caused by circFNDC3B depletion. CircFNDC3B may be a potential therapeutic target for oral squamous cell carcinoma (Yang et al., 2021).

### 4.2 Thyroid cancer

Circ_0067934 has been found to play a pro-carcinogenic role in a variety of cancers, and it was found that in thyroid cancer, knockdown of circ_0067934 enhanced Fe2+, intracellular iron and ROS levels and promoted the development of ferroptosis, while inhibition of miR-545-3p and overexpression of SLC7A11 reversed this effect. circ_0067934 competitively binding to miR-545-3p upregulates SLC7A11 to inhibit ferroptosis, promotes thyroid cancer development and advances thyroid cancer treatment (Wang et al., 2021b).

### 4.3 Esophageal cancer

CircBCAR3 was found to interact with miR-27a-3p to upregulate transportin 1 (TNPO1) expression, promote esophageal cancer cell proliferation, migration, invasion and ferroptosis, and play an oncogenic role in esophageal cancer by promoting esophageal tumorigenesis and metastasis in mice (Xi et al., 2022).

CircPVT1 also plays a role in esophageal cancer as a circular RNA. CircPVT1 sponge miR-30a-5p targets and binds frizzled class receptor 3 (FZD3), affects the expression levels of p-β-catenin, GPX4 and SLC7A11, inhibits cellular ferroptosis, affects esophageal cancer 5-FU chemotherapy sensitivity and promotes the development of esophageal cancer. This could be a potential target for esophageal cancer therapy (Yao et al., 2021).

### 4.4 Lung cancer

In China, where the incidence and mortality of lung cancer are high (Wang et al., 2020). Showed that downregulation of circDTL promotes ferroptosis in non-small cell lung cancer cells. Mechanistically, circDTL targets GPX4 via the circDTL sponge miR1287-5p to inhibit the onset of cellular ferroptosis, which in turn exerts its tumorigenic effects (Shanshan et al., 2021).

CircP4HB was found to be a novel ferritinase inhibitor in lung adenocarcinoma. CircP4HB affects GSH synthesis by targeting SLC7A11 through the sponge miR-1184, inhibiting ferroptosis and promoting tumor cell growth. circP4HB may be used as a molecular target for the treatment of lung cancer in the future (Pan et al., 2022).

Similarly, the exosome circRNA_101093 (cir93) plays an important role in ferroptosis and regulates the development of lung adenocarcinoma. Overexpression of cir93 inhibited erastin-induced ferroptosis and reduced lipid peroxidation to inhibit ferroptosis occurrence by upregulating fatty acid binding protein 3 (FABP3) expression. In addition, cir93 interacted with FABP3 thereby regulating NAT (a product of taurine and AA) to inhibit ACSL4, LPCAT3 and phospholipid transfer protein (PLTP) to reduce AA impeding ferroptosis progression. This may hold promise for future lung adenocarcinoma treatment (Zhang et al., 2022a).

In lung cancer, circSCN8A overexpression increased Fe2+, ROS and MDA levels and decreased GSH levels. Western blotting demonstrated that circSCN8A overexpression downregulated SLC7A11 and GPX4 protein levels and promoted ferroptosis, which was reversed by the addition of Ferrostatin-1 (Fer-1), an ferroptosis inhibitor. In addition, circSCN8A bound miR-1290 to enhance ACSL4 expression to promote lung cancer proliferation and metastasis and inhibit ferroptosis. Thus, circSCN8A may be a promising therapeutic target against lung cancer (Liu et al., 2023).

### 4.5 Gastric cancer

Jiang et al. (2022a) found that circ_ 0000190 targeted the tumor suppressor zinc and ring finger 3 (ZNRF3) by competitively binding to miR-382-5p, inhibited gastric cancer cell proliferation, migration and invasion, and stimulated ferroptosis to occur, ultimately leading to inhibition of gastric cancer progression. Upregulation of circ_0000199 or ZNRF3 expression may be an effective strategy for the treatment of GC.

The role of Circ_0008035 in gastric cancer has been confirmed by several studies. However, how Circ_0008035 regulates ferroptosis has not been reported yet. Li et al. (2020) found knockdown of circ_0008035 could promote ferroptosis, and circ_0008035 acted as a sponge for miR-599 and suppressed the expression of its target gene eukaryotic translation initiation factor 4A1 (EIF4A1). Overexpression of EIF4A1 reversed the effects of knockdown of circ_0008035 on iron accumulation, lipid peroxidation and mitochondrial membrane potential in gastric cancer cells. In conclusion, the circ_0008035/miR-599/EIF4A1 axis could regulate the occurrence of ferroptosis in gastric cancer cells and thus affect gastric cancer progression. Moreover, Circ0008035 was found to be another pathway regulating cellular ferroptosis affecting gastric cancer progression. Gao et al. found that circ0008035 increased E2F transcription factor 2 (E2F2) expression by acting as a ceRNA for miR-302a. Circ0008035 inhibited dextromethorphan-induced ferroptosis, and overexpression of miR-302a or knockdown of E2F transcription factor 7 (E2F7) effectively reversed this effect (Gao and Wang, 2023). In other words, circ0008035 can affect ferroptosis regulation of tumorigenesis through two independent parallel pathways, which provides a viable therapeutic option for gastric cancer.

CircRPPH1 also plays a regulatory role in gastric cancer. CircRPPH1 as a miR-375 sponge positively regulates SLC7A11 expression and has been shown to be a direct target of miR-377 in cancer, thereby regulating cellular ferroptosis. Knockdown of circRPPH1 promoted GSH synthesis, caused lipid peroxidation, induced cellular ferroptosis, and inhibited gastric cancer progression. And inhibition of miR-375 or overexpression of SLC7A11 partially reversed this effect. This may be a future research direction for gastric cancer treatment (Liu et al., 2022b).

### 4.6 Breast cancer

Breast cancer is the most prevalent cancer in women. In breast cancer, circGFRA1b enhances the activity of AIFM2 by binding to miR-1228, and the expression of GPX4 and the GSH/GSSG ratio are upregulated. In other words, HER2-positive breast cancer cells can inhibit ferroptosis by activating two pathways. Therapies targeting these two pathways may be able to inhibit breast cancer progression (Bazhabayi et al., 2021).

### 4.7 Prolactinoma

CircOMA1 is a pro-oncogene. CircOMA1 was first identified to regulate ferroptosis to affect proliferation of prolactinomas. CircOMA1 acts as a miR-145-5p sponge to regulate the expression level of glutamate-cysteine ligase, modifier subunit (GCLM), inhibit ferroptosis and promote tumor development. Moreover, circOMA1 also impedes ferroptosis by upregulating the expression of NRF2, GPX4 and xCT, key genes of ferroptosis. In other words, circOMA1 inhibits ferroptosis and promotes the development of prolactinoma through two parallel and independent pathways, which provides a new pathway for the treatment of prolactinoma (Wu et al., 2023).

### 4.8 Hepatocellular carcinoma

In hepatocellular carcinoma, the involvement of the circRNA cIARS in the regulation of ferroptosis through the inhibition of ALKBH5 was demonstrated by rescue experiments to influence cancer progression and represents a key point in the treatment of hepatocellular carcinoma (Liu et al., 2020).

SLC7A11 is a key regulator of iron uptake by cancer cells. Downregulation of the circ0097009 gene promotes iron uptake by hepatocellular carcinoma cells through two independent pathways: system xc- and GPX4. This study confirms that circ0097009 and miR-1261 competitively bind to target SLC7A11 to mediate hepatocellular carcinoma progression through regulation of ferroptosis (Lyu et al., 2021).

In addition, knockdown of circIL4R in hepatocellular carcinoma upregulated cellular iron level and oxidative stress and promoted erastin-induced ferroptosis. CircIL4R competitively bound to miR-541-3p, and inhibition of miR-541-3p replied to the tumor suppression caused by circIL4R knockdown and activated ferroptosis. GPX4 plays an important role in regulating ferroptosis as a target gene of miR-541-3p. In conclusion, circIL4R inhibits ferroptosis through miR-541-3p/GPX4 axis and promotes hepatocellular carcinogenesis (Xu et al., 2020).

### 4.9 Colorectal cancer

Circ_0007142 is overexpressed in colorectal cancer and targets glycerophosphodiester phosphodiesterase domain containing 5 (GDPD5) through binding to miR-874-3p, triggering the onset of ferroptosis and thus inhibiting the progression of colorectal cancer. In conclusion, demonstrated that circ_0007142 can be involved in the regulation of ferroptosis and colorectal cancer progression. Circ_0007142 may be a ferritinase marker for the treatment of colorectal cancer (Wang et al., 2021b).

Similarly, circSTIL plays an important role in colorectal cancer. Knockdown of circSTIL induces cellular Fe2+ and lipid ROS accumulation, activates cellular ferroptosis, and inhibits colorectal cancer cell proliferation. In addition, circSTIL targets SLC7A11 by competitively binding to miR-431 to regulate ferroptosis, providing a new target for the treatment of colorectal cancer (Li et al., 2023).

### 4.10 Rectal cancer

Numerous studies have shown that circABCB10 plays an important role in a variety of cancers. However, the reports of circABCB10 on ferroptosis are still relatively few and deserve further consideration and research. Xian et al. (2020) found that knockdown of circABCB10 *in vitro* stimulated ferroptosis in colorectal carcinoma cells. CircABCB10 acts as a miR-326 sponge competing for binding to C-C motif chemokine ligand 5 (CCL5), regulating the expression level of CCL5, and inhibition of miR-326 partially reversed circABCB10-induced ferroptosis. In conclusion, knockdown of circABCB10 promotes ferroptosis in colorectal carcinoma cells through miR-326/CCL5 axis and inhibits the further development of colorectal cancer, providing a new perspective on the progression of colorectal cancer.

### 4.11 Cervical cancer

In addition, circLMO1 inhibits the growth of cervical cancer cells by adsorbing miR-4192 to repress the target gene ACSL4, which promotes ferroptosis in cervical cancer cells. In conclusion, circLMO1 is expressed at low levels in cervical cancer, and overexpression of circLMO1 may be useful in cervical cancer treatment (Ou et al., 2022).

Similarly, circACAP2 plays a ceRNA role in cervical cancer cells, acting directly with miR-193a-5p to target GPX4, a key protein for ferroptosis, to inhibit ROS accumulation and intracellular iron levels. This suggests that circACAP2 inhibits ferroptosis and promotes the malignant progression of cervical cancer (Liu and Chen, 2022). In conclusion, circRNA related ferroptosis is involved in the regulation of cancer progression, which provides a new idea for cancer targeted therapy. However, the current understanding of its mechanism is not sufficiently comprehensive and thorough. Therefore, further study is urgently required.

### 4.12 Ovarian cancer

Cisplatin chemotherapy resistance (DDP) is one of the modalities of ovarian cancer treatment, however, it was found that knockdown of circSnx12 stimulated ferroptosis production and enhanced cisplatin sensitivity. In addition, circSnx12 inhibits ferroptosis and promotes ovarian cancer progression by targeting SLC7A11 through the sponge miR-194-5p. In conclusion, circSnx12 can regulate ferroptosis and chemoresistance, which are crucial for the treatment of ovarian cancer (Qin et al., 2023).

### 4.13 Bladder cancer

CircST6GALNAC6 plays an oncogene in bladder cancer, however, whether circST6GALNAC6 has a regulatory role on ferroptosis is currently unknown. Wang et al. first found that circST6GALNAC6 could directly bind to HSPB1, blocking erastin-induced phosphorylation of HSPB1 and promoting cellular ferroptosis. CircST6GALNAC6 could also inhibit HSPB1 to suppress bladder cancer cell proliferation and promote cellular ferroptosis by activating the P38 MAPK signaling pathway (Wang L et al., 2022; Wang S et al., 2022b).

### 4.14 Endometrial cancer

It was found that in endometrial cancer, circRAPGEF5 acts with RNA binding fox-1 homolog 2 (RBFOX2) to block the binding of RBFOX2 to pre-mRNA and inhibit TFRC expression. Overexpression of circRAPGEF5 leads to a reduction in the pool of unstable iron and a reduction in lipid peroxidation in endometrial cancer cells, resulting in resistance to ferroptosis. These findings may validate the emerging mechanism by which circRNA mediates ferroptosis through regulation of selective splicing (Zhang X. et al, 2022).

### 4.15 Glioma

In glioma, circCDK14 was found to reduce the likelihood of ferroptosis occurring in glioma cells by regulating the expression levels of platelet derived growth factor receptor alpha (PDGFRA), which is closely associated with poor prognosis in glioma patients and promotes the malignant progression of gliomas (Chen et al., 2022). CircLRFN5 can mediate paired related homeobox 2 (PRRX2) via the ubiquitin proteasome pathway, and in gliomas, PRRX2 activates transcriptional upregulation of GTP cyclohydrolase 1 (GCH1) expression, a substance that inhibits ferroptosis by producing the antioxidant tetrahydrobiopterin (BH4). This implies that circLRFN5 can mediate ferroptosis in glioma (Jiang et al., 2022b).

## 5 Conclusion and future perspective

Tumors are a disease type commanding substantial interest worldwide. The diagnostic and therapeutic targets of tumors have been a hot topic of research in this field. In recent years, various studies have confirmed that ferroptosis can bind non-coding RNAs to regulate tumors. MiRNAs and lncRNAs have been extensively studied. As an emerging ncRNA, circRNAs have been shown to be associated with ferroptosis.

Overall, ferroptosis is mainly regulated through four independent parallel molecular pathways (Figure 4). The first is the glutathione pathway. For example, the circRNA circACAP2 promotes the malignant progression of cervical cancer by competitively binding to and targeting the GPX4 ferroptosis key protein and inhibiting the onset of ferroptosis (Liu J. et al, 2022). The second pathway is the ubiquinone pathway (NADPH-FSP1-CoQ10). CircGFRA1 binding to miR-1228 enhances FSP1 expression to inhibit ferroptosis and promote the development of HER2-positive breast cancer (Bazhabayi et al., 2021). Third, in the GCH1-BH4 pathway, circLRFN5 regulates GCH1-activated ferroptosis via PRRX2 and inhibits glioblastoma progression (Jiang et al., 2022b). Moreover, ACSL4 and LPCAT3 mediate the depletion of PUFA-PLs. The circular RNA circLMO1 regulates ACSL4 by triggering miR-4291, which induces ferroptosis and suppresses cervical cancer malignancy (Ou et al., 2022).

However, research into the involvement of circRNAs in the regulation of ferroptosis in cancer development is relatively sparse and still in its infancy. Ferroptosis in tumor biology is complex and highly context-dependent with respect to treatment. Exploring the pathogenesis of ferroptosis and binding to circRNAs in various tumors is a direction for our future research.
